# *N*-Acetylcysteine prevents amyloid-β secretion in neurons derived from human pluripotent stem cells with trisomy 21

**DOI:** 10.1038/s41598-021-96697-7

**Published:** 2021-08-30

**Authors:** Hiromitsu Toshikawa, Akihiro Ikenaka, Li Li, Yoko Nishinaka-Arai, Akira Niwa, Akira Ashida, Yasuhiro Kazuki, Tatsutoshi Nakahata, Hiroshi Tamai, David W. Russell, Megumu K. Saito

**Affiliations:** 1 Department of Clinical Application, Center for iPS Cell Research and Application, Kyoto University, 53 Shogoin-Kawahara-cho, Sakyo-ku, Kyoto, 606-8507 Japan; 2 Osaka Medical and Pharmaceutical University, Takatsuki, 5690801 Japan; 3 Social Welfare Organization “SAISEIKAI” Imperial Gift Foundation Inc., Saiseikai Suita Hospital, Suita, 5640013 Japan; 4 Division of Hematology, School of Medicine, University of Washington, Seattle, WA 98195 USA; 5 Department of Human Health Sciences, Graduate School of Medicine, Kyoto University, Kyoto, 6068507 Japan; 6 Chromosome Engineering Research Center, Tottori University, Tottori, Japan; 7 Division of Genome and Cellular Functions, Department of Molecular and Cellular Biology, School of Life Science, Faculty of Medicine, Tottori University, Tottori, Japan; 8 Institute for Developmental Brain Research, Osaka Medical and Pharmaceutical University, Takatsuki, 5690801 Japan

**Keywords:** Dementia, Induced pluripotent stem cells, Developmental disorders

## Abstract

Down syndrome (DS) is caused by the trisomy of chromosome 21. Among the many disabilities found in individuals with DS is an increased risk of early-onset Alzheimer's disease (AD). Although higher oxidative stress and an upregulation of amyloid β (Aβ) peptides from an extra copy of the *APP* gene are attributed to the AD susceptibility, the relationship between the two factors is unclear. To address this issue, we established an in vitro cellular model using neurons differentiated from DS patient-derived induced pluripotent stem cells (iPSCs) and isogenic euploid iPSCs. Neurons differentiated from DS patient-derived iPSCs secreted more Aβ compared to those differentiated from the euploid iPSCs. Treatment of the neurons with an antioxidant, N-acetylcysteine, significantly suppressed the Aβ secretion. These findings suggest that oxidative stress has an important role in controlling the Aβ level in neurons differentiated from DS patient-derived iPSCs and that N-acetylcysteine can be a potential therapeutic option to ameliorate the Aβ secretion.

## Introduction

Down syndrome (DS) is the chromosome abnormality defined by an extra copy of chromosome 21. DS develops various complications such as neurological, skeletal, cardiovascular, and immunological defects^1^, but is perhaps best known for being the most common genetic cause of mental retardation and intellectual disability^2^, occurring at a rate of 1 in 800 to 1000 births^3,4^. Chromosome 21 contains genes related to neurodegenerative diseases and oxidative stress^5^, and research on the involvement of these genes on the pathophysiology of DS is underway^6^.

Consistent with the neurological complications and intellectual disabilities is that individuals with DS have a higher susceptibility to early onset Alzheimer's disease (AD)^7,8^. One of the hypothesized reasons is the existence of an extra copy of *amyloid precursor protein (APP)* gene located on chromosome 21. APP is a precursor of amyloid-beta (Aβ), and the extra copy increases the expression of *APP* and subsequent production of Aβ^9,10^. Since AD-like pathological lesions such as Aβ deposition can be recognized in individuals with DS younger than 40 years old and symptoms of cognitive impairment due to AD exponentially increases from this age, DS has been regarded as a “young model” of AD^1,6,11^. Connecting AD and DS is Aβ42, which plays an important role in the pathogenesis of AD and may also affect the cognitive function seen in DS^12,13^.

It has been suggested that the brains of individuals with DS are exposed to oxidative stress^14–16^. Oxidative stress plays a central role in neurogenic changes in DS^4,17^. In animal studies, the administration of antioxidants from the fetal period increases the number of cells in the hippocampus^18^, indicating that early intervention to circumvent the effect of oxidative stress may be important to improve the neurological prognosis. Oxidative stress in DS is attributed to several genes on chromosome 21, including an extra copy of *superoxide dismutase 1 (SOD1)*^19^. Oxidative stress is enhanced because of the accumulation of H_2_O_2._ The overexpression of *SOD1* produces a large amount of H_2_O_2_ that cannot be catalyzed because the copy number of *catalase* is normal^19^. However, in experiments using DS model mice, oxidative stress was increased even when only two copies of *SOD1* existed^20^. Therefore, although oxidative stress is caused by the overexpression of *SOD1*, other factors are also involved.

The relationship between oxidative stress and Aβ is complicated. Oxidative stress may be involved in Aβ production^21^, and Aβ42 oligomers are known to produce reactive oxygen species (ROS) and have neurotoxicity^22–24^. Accurate evaluation of the regulatory role of oxidative stress on the production of Aβ production is therefore important for establishing an appropriate therapeutic strategy for DS-related neuronal degeneration.

In this study, we differentiated induced pluripotent stem cells (iPSCs) derived from individuals with DS (D-iPSCs) into neurons (D-iNs) and measured their Aβ production. Secreted Aβ was increased in D-iNs, and the amount of Aβ was reduced by a high-dose administration of the antioxidant N-acetylcysteine (NAC). Similar results were obtained from a human embryonic stem cell (ESC) line with trisomy 21. Although many animal models of DS have been constructed, no model animal can completely trace the symptoms occurring in humans. Our study succeeded in establishing an in vitro human model to evaluate the effect of oxidative stress on neurons with trisomy 21.

## Results

### Conversion of D-iPSCs into neurons

**Figure 1:**
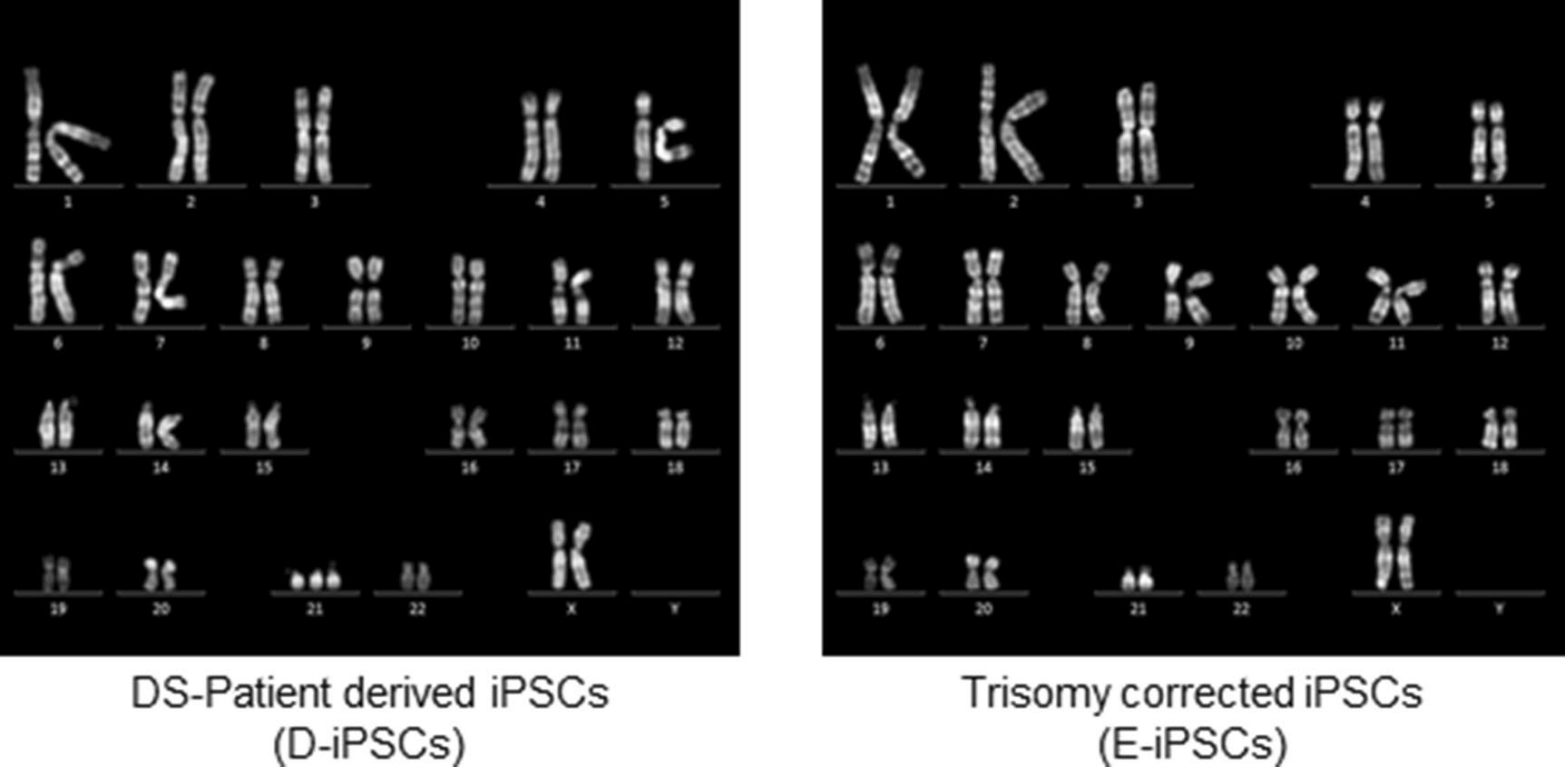
Karyotype analysis of the iPSC clones.

**Figure 2:**
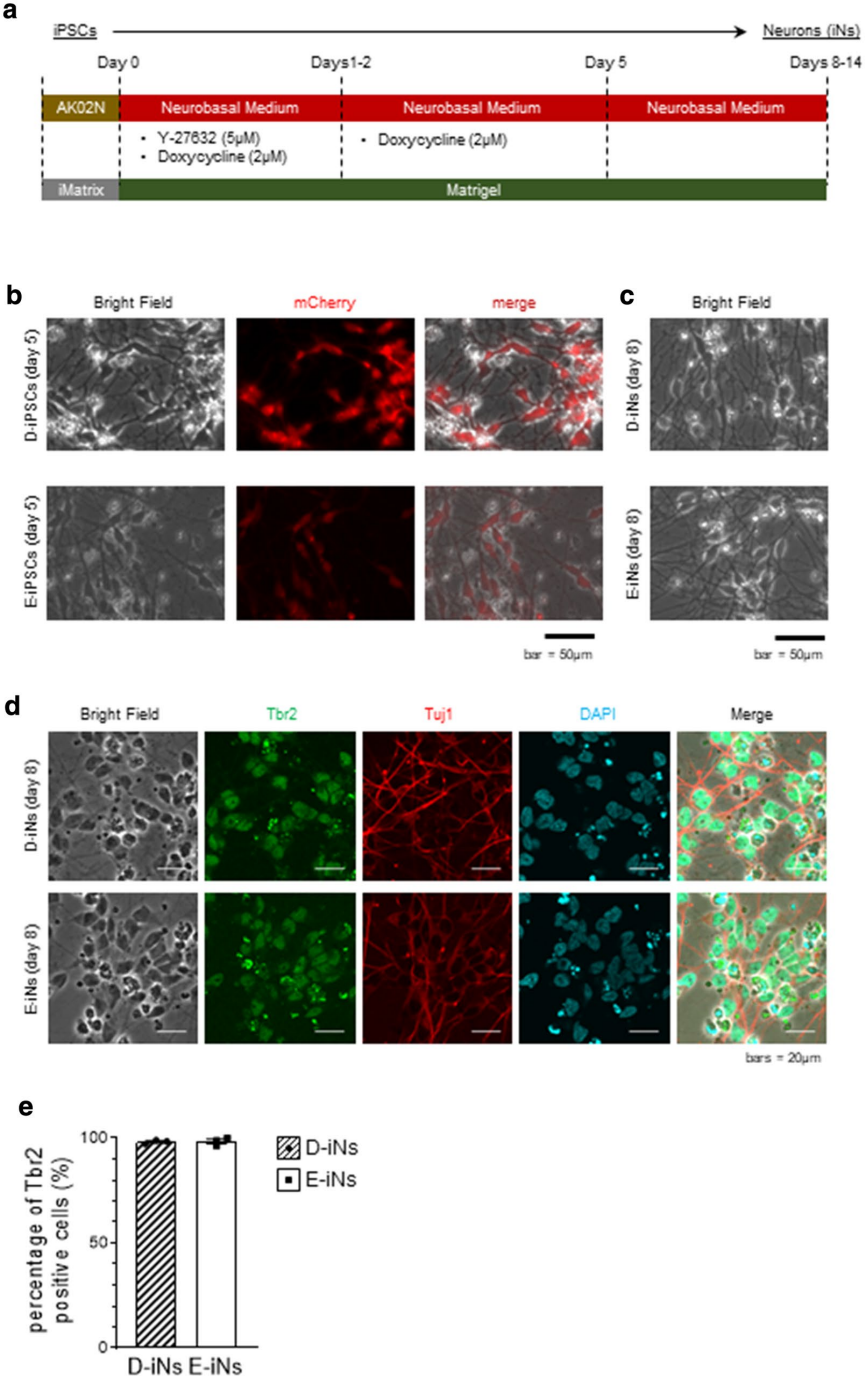
Neuronal differentiation with over-expression of *NGN2*. **(a)** Schematic of the neuronal differentiation using the doxycycline-inducible *NGN2* expression system. **(b)** mCherry expression at day 5. **(c)** Bright-field images of neuronal cells on day 8. **(d)** Immunostaining images of neuronal cells on day 8. **(e)** Quantification of the Tbr2-positive cells in **(d)**. Data are means ± SEM from three independent experiments.

To precisely evaluate the phenotype of iNs, we used D-iPSCs and an isogenic euploid control clone established from the D-iPSCs (E-iPSCs)^25^ (Fig. 1). These iPSCs were then directly converted into neuronal lineage cells by the transient overexpression of *Neurogenin 2* (*NGN2*) gene^26,27^ (Fig. 2a). For this, we incorporated a doxycycline-inducible expression vector encoding *NGN2* and mCherry fluorescent protein into the iPSC clones and treated the clones with doxycycline for 5 days (Fig. 2a). At day 5, almost all cells were positive for mCherry (Fig. 2b) and showed a compatible morphological appearance with neurons, such as elongated neurites (Fig. 2b,c). At day 8, almost all cells expressed an intermediate neuronal progenitor cell, marker Tbr2, and were positive for a neuron-specific human tubulin protein, Tuj1 (Fig. 2d,e). These observations confirmed the neuronal differentiation of D-iPSCs and E-iPSCs.

### D-iNs secrete a larger amount of Aβ

**Figure 3:**
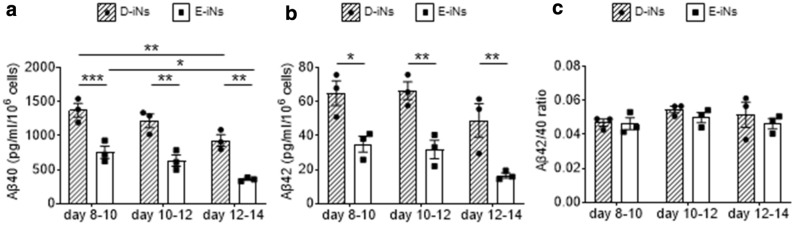
Aβ secretion from iNs at different periods. **(a,b)** Amount of secreted Aβ40 **(a)** and Aβ42 **(b)** from iNs at the indicated periods. (**c)** The Aβ40/Aβ42 ratio at each period. ELISA results obtained from technical duplicates and biological triplicates are shown. Data are means ± SEM; ***, p < 0.001; **, p < 0.01; *, p < 0.05; two-way ANOVA test followed by Bonferroni’s multiple comparison test **(a,b)**. Two-way ANOVA found no significance (interaction p value = 0.8188) in **(c)**.

We next evaluated the secretion of Aβ peptides Aβ40 and Aβ42 from iNs. β-secretase-1 (beta-site APP cleaving enzyme; BACE1) and γ-secretase cleave APP to produce Aβ peptides, and Aβ peptides exert neurotoxicity through various proposed mechanisms^28–31^. In particular, Aβ42 plays an essential and important role in all AD. Aβ42 is highly hydrophobic and has high aggregation properties. Additionally, a high Aβ42/Aβ40 ratio is associated with the formation of amyloid plaques found in familial AD patients^12,32^. We therefore evaluated the amount of Aβ secretion from D-iNs and E-iNs, finding the amount of secreted Aβ40 and Aβ42 was higher in D-iNs at all times observed (Fig. 3a,b). However, the Aβ42/Aβ40 ratio was not different between the two iN types (Fig. 3c). These data indicated that D-iNs are predisposed to secrete more Aβ protein.

### Oxidative stress affects the amount of secreted Aβ from D-iNs

**Figure 4:**
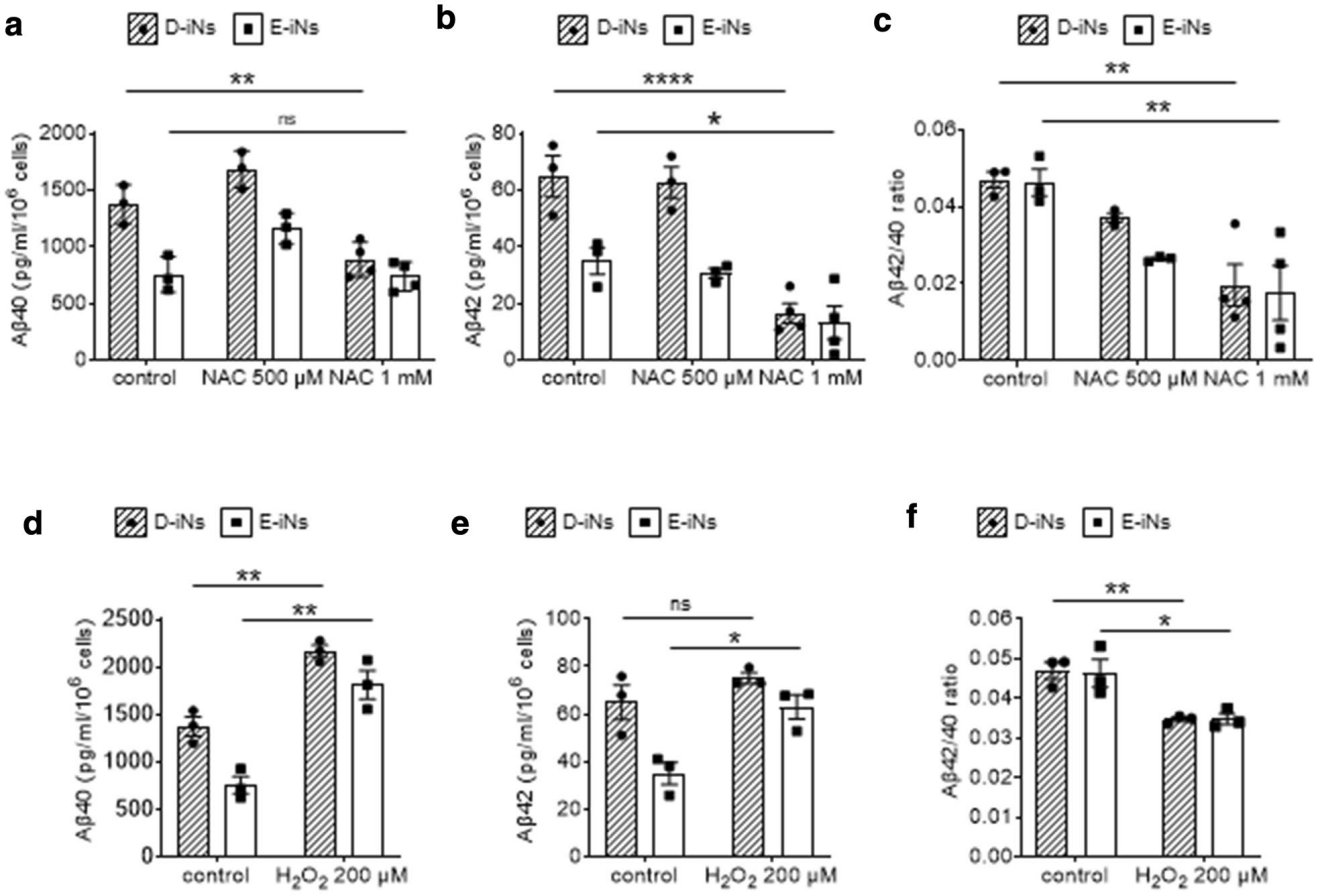
Effects of an oxidant or anti-oxidant on Aβ secretion from iNs. (**a–c)** Amount of secreted Aβ40 **(a)** and Aβ42 **(b)** from iNs and the Aβ40**/**Aβ42 ratio **(c)** at day 10. NAC at the indicated concentrations was added on day 8. (**d–f)** Amount of secreted Aβ40 **(d)** and Aβ42 **(e)** from iNs and the Aβ40**/**Aβ42 ratio **(f)** at day 10. H_2_O_2_ (200 μM) was added on day 8. ELISA results obtained from technical duplicates and biological triplicates are shown. Data are means ± SEM; ***, p < 0.001; **, p < 0.01; *, p < 0.05; two-way ANOVA test followed by Bonferroni’s multiple comparison test **(a–c)** or Student’s t-test **(d–f)**. Control data in **(a,d)**, **(b,e)**, and **(c,f)** are the same as the corresponding data for days 8–10 in Fig. 3a–c, respectively.

The brains of individuals with DS are exposed to high oxidative stress. The deposition of Aβ increases oxidative stress^33–35^, and the existence of an extra copy of *superoxide dismutase 1 (SOD1)* causes the overproduction of H_2_O_2_. Consistently, antioxidants and catalase show neuroprotective effects in individuals with DS and model mice^14,18^. Therefore, we investigated the effect of an antioxidant and an oxidant on the amount of Aβ secretion from iNs. NAC treatment decreased Aβ secretion at the highest dose (Fig. 4a,b). On the contrary, H_2_O_2_ treatment significantly increased the secretion of Aβ protein from both D-iNs and E-iNs (Fig. 4d,e). Interestingly, both NAC and H_2_O_2_ treatment decreased the Aβ42/Aβ40 ratio (Fig. 4c,f). These findings show oxidative stress positively correlates with the secretion of Aβ protein from iNs.

### Effect of oxidative stress on the expression of *APP*

**Figure 5:**
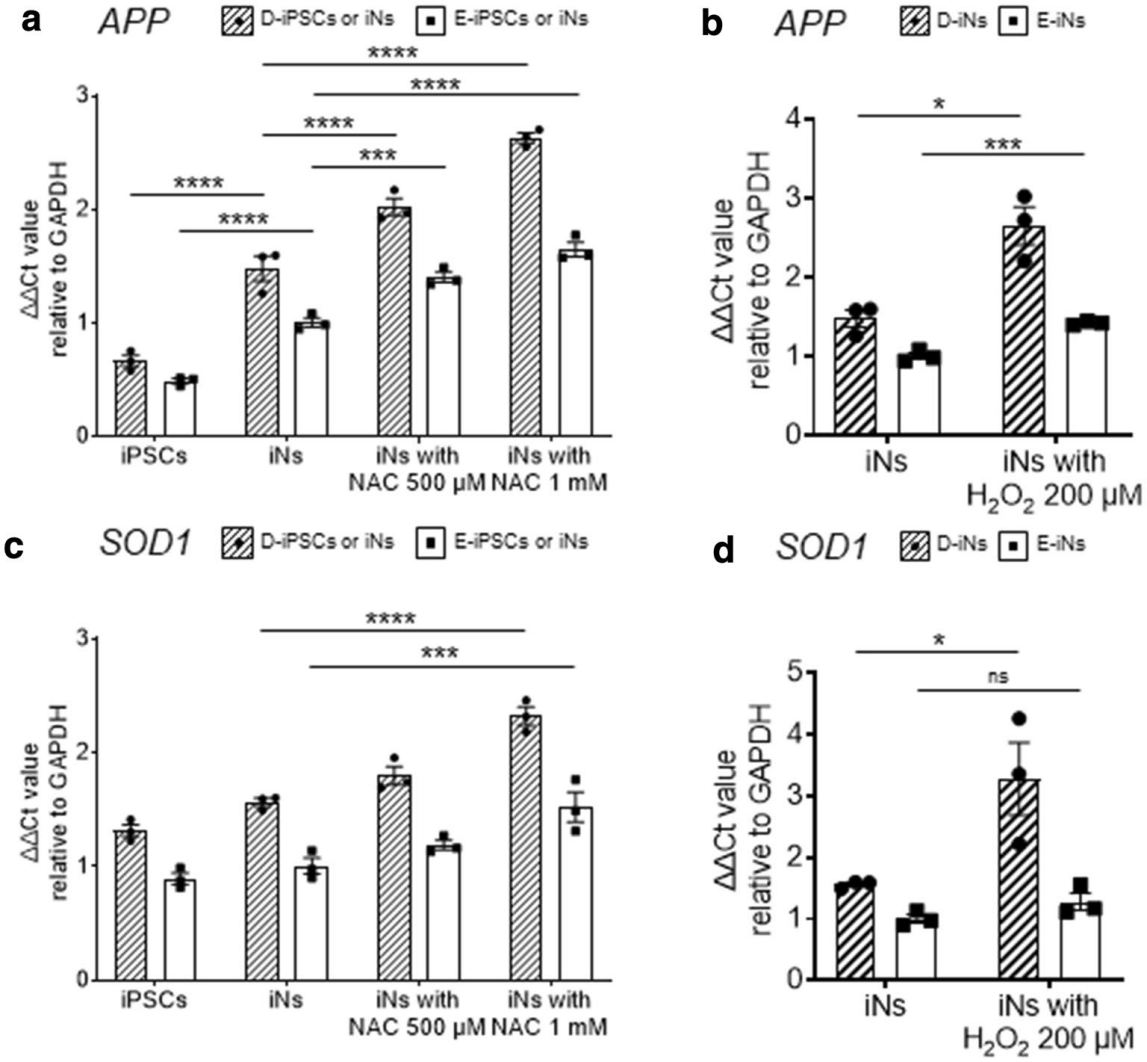
Effects of an oxidant or anti-oxidant on the expression of *APP* and *SOD1*. **(a–d)** Expression of *APP* **(a,b)** and *SOD1* **(c,d)** evaluated by the ΔΔCt method relative to GAPDH. Data were converted into 2^-ΔΔCt^ values and plotted. NAC or H_2_O_2_ at the indicated concentrations were added on day 8, and the samples were collected on day 10. Data are means ± SEM from three independent experiments; ****, p < 0.0001; ***, p < 0.001; **, p < 0.01; two-way ANOVA test followed by Dunnett’s multiple comparison test **(a,c)** or Student’s t-test **(b,d)**. Data are normalized to the mean expression levels of control E-iNs. Data for iNs in **(a)** and **(b)** and in **(c)** and **(d)** are the same.

We next examined whether oxidative stress affects the expression of *APP* in iNs. *APP* was upregulated during the neuronal differentiation of D-iPSCs, as expected (Fig. 5a). H_2_O_2_ treatment upregulated the expression of *APP* gene both in D-iNs and E-iNs, but the effect was more prominent in D-iNs (Fig. 5b). NAC treatment also upregulated *APP* expression (Fig. 5a), indicating that the NAC effect is related to posttranscriptional modifications. The expression of *SOD1* was upregulated by treatment with H_2_O_2_ or NAC (Fig. 5c,d).

### Verification with another isogenic PSC pair

**Figure 6:**
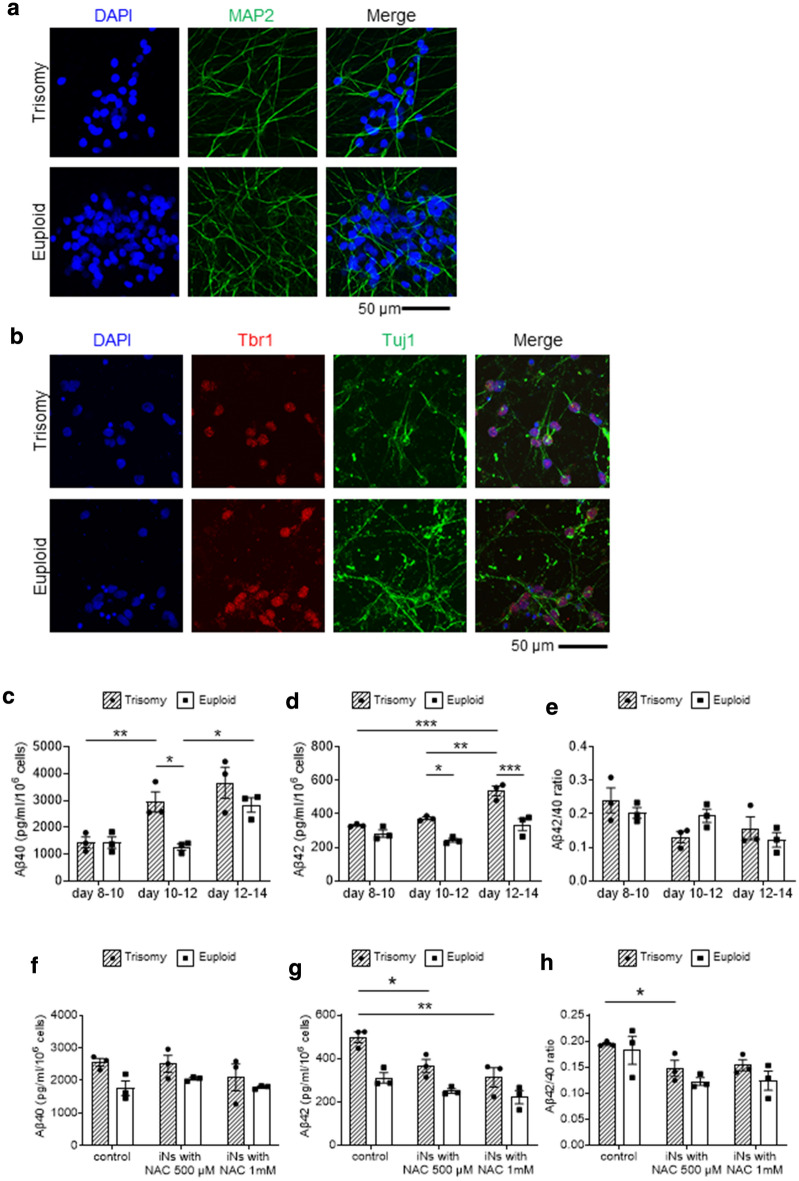
Verification with another isogenic PSC pair. **(a,b)** Immunostaining of ESC-derived neurons on day 14. (**c,d)** Amount of secreted Aβ40 **(c)** and Aβ42 **(d)** from ESC-derived neurons at the indicated periods. (**e)** The Aβ40/Aβ42 ratio at each period. (**f–h)** Amount of secreted Aβ40 **(f)** and Aβ42 **(g)** from iNs and the Aβ40**/**Aβ42 ratio **(h)** at day 10. NAC at the indicated concentrations was added on day 8. Data are means ± SEM from three independent experiments; ***, p < 0.001; **, p < 0.01; *, p < 0.05; two-way ANOVA test followed by Tukey’s Multiple Comparison Test. Two-way ANOVA found no significance in **(e,f)**: interaction p values were 0.1242 **(e)** and 0.5453 **(f)**.

To confirm that the increased Aβ secretion and the inhibitory effect of high-dose NAC are not a clone-specific phenomenon, we tested the reproducibility of the results using another isogenic PSC pair. We used KhES1, a euploid human ESC line, and a subclone of KhES1 in which chromosome 21 was artificially inserted to make trisomy 21^36^. We introduced a doxycycline-inducible *NGN2* expression vector into these clones and induced neuronal differentiation. Both clones showed good NGN2-dependent differentiation properties and differentiated into neurons expressing Tuj1, MAP2 and Tbr1 (Fig. 6a,b). As in the case with the DS-derived clones, Aβ secretion was increased in a time-dependent manner and higher in the trisomy clone after day 12 (Fig. 6c–e). NAC administration reduced the production of Aβ42 but had no significant effect on Aβ40 (Fig. 6f–h). These results indicate that the increased secretion of Aβ from trisomy 21 and the inhibitory effect of high-dose NAC are not clone- dependent.

## Discussion

Here we investigated the effect of trisomy 21 on neuronal Aβ by using trisomy 21 iPSCs and their isogenic euploid control and differentiating them into neurons. Aβ was produced early after initiating the direct conversion and higher in D-iNs. In addition, the Aβ production was reduced by using an antioxidant, NAC.

The brains of individuals with DS show pathological changes similar to those of AD patients^11,37^. In familial AD, the proportion of Aβ42 increases from the early stage due to genetic abnormalities of PSEN1 or PSEN2. A relative increase of Aβ42 to Aβ40 has been considered a risk of synaptic dysfunction^12,22,29^. On the other hand, in DS, the total amount of both Aβ40 and Aβ42 increases due to the increased copy number of *APP* caused by extra chromosome 21. Therefore, the Aβ42/Aβ40 ratio is considered unchanged, especially in the early stage^38^, which is consistent with our findings.

Treatment with various antioxidants has been tried against the increased oxidative stress in DS. However, the effects were often partial or limited in animal models^39–42^. In the present study, we found that NAC treatment dose-dependently reduced the production of Aβ from iNs, which is consistent with previous studies on DS and AD. NAC is a precursor of glutathione peroxidase and is known as an antioxidant that can prevent the enhanced death due to oxidative stress of neurons derived from D-iPSCs^17^. NAC treatment was also seen to improve the cognitive memory behavior of AD model mice and rats^43,44^ and to suppress neuroinflammation^45^. Therefore, NAC itself can reduce oxidative stress in the whole brain and exert a neuronal cell protective effect. Adding our study gives further argument to NAC improving the prognosis of the cognitive function of DS.

Although the addition of NAC suppressed the secretion of Aβ, it did not down-regulate the expression of *APP*. This finding suggests that the anti-oxidative stress effect of NAC may affect the cleavage of *APP* protein post-transcriptionally. Aβ production is known to be affected by oxidative stress, and increased oxidative stress up-regulates *BACE1* expression^46^ and *PSEN1* expression in lipid rafts^47,48^. Since PSEN1 is an active center of γ-secretase, the activity of γ-secretase may also be increased by oxidative stress^21^. In addition, the β and γ secretase-dependent processing of *APP* is promoted by α-synuclein^49^. These reports and our data indicate that, in addition to an extra copy of *APP*, the brains of individuals with DS are exposed to an unfavorable environment where Aβ production is enhanced due to increased oxidative stress.

In anticipation of the antioxidant effect of NAC, clinical trials have been conducted on various neuropsychiatry diseases and neurodegenerative diseases^50–52^. In this study, we showed the effect of NAC in iNs, suggesting that the neuroprotective effect of NAC can be expanded to diseases associated with Aβ and oxidative stress. However, our study is based upon a relatively small number of control and DS cases and it will be important for this work to be replicated using larger sample sizes.

## Materials and methods

### Ethics statement

This study was approved by the Ethics Committee and the recombinant DNA Experiments Safety Committee of Kyoto University. The use of human ESCs was approved by the Ministry of Education Culture, Sports, Science and Technology of Japan (MEXT). All methods were performed in accordance with the relevant guidelines and regulations. Informed consent was obtained from the legal guardians of the DS patient.

### iPSC clones and introduction of the doxycycline-inducible NGN2 expression vector

We used an iPSC clone obtained from a DS patient and a trisomy-corrected isogenic clone generated from the trisomy clone as previously described^25^. The source fibroblasts of the iPSCs were obtained from the Coriell Institute for Medical Research (AG06892). As another isogenic pair, we used an euploid ESC line KhES1 and trisomy KhES1 subclone with chromosome 21 artificially introduced^36,53^. The original euploid KhES1 clone was kindly provided by Hirofumi Suemori (Institute for Frontier Medical Sciences, Kyoto University, Kyoto, Japan).

For the generation of iPSC-derived neurons, we took advantage of the doxycycline-induced *NGN2* expression system^26,27^. We constructed a plasmid vector encoding human NGN2 cDNA under the tetracycline-inducible promoter (*TetO::NGN2-IRES-mCherry*). The piggyBac backbone vector, PB-TAC-ERN, was a gift from Dr. Knut Woltjen (Addgene plasmid #80475; http://n2t.net/addgene:80475; RRID: Addgene_80475)^26^. We selected subclones showing high differentiation ability after the antibiotic selection with G418 disulfate (FUJIFILM Wako Pure Chemical Corporation, Osaka, Japan).

### Neuronal induction and sample taking

Neuronal induction by doxycycline-inducible *NGN2* expression was performed as previously described^27^. Briefly, on day 0, iPSCs were dissociated with TrypLE Select (GIBCO, Thermo Fisher Scientific, MA, USA) and disseminated on a mixed coating of poly-l-lysine (final 0.0002% v/w, Merck, Darmstadt, Germany), and Matrigel (final 2% v/v, Corning, NY, USA). The disseminated iPSCs were cultured in Neurobasal Medium (GIBCO, Thermo Fisher Scientific, MA, USA) supplemented with 0.5% B27 without vitamin A (GIBCO, Thermo Fisher Scientific, MA, USA), 1 × Glutamax (GIBCO, Thermo Fisher Scientific, MA, USA), 2 mg/mL doxycycline hydrochloride (FUJIFILM Wako Pure Chemical Corporation, Osaka, Japan), and 5 mM Y-27632 (Nacalai-Tesque, Kyoto, Japan). Y-27632 was withdrawn on day 1 or 2. On day 5, we exchanged culture media for new media without doxycycline hydrochloride. To evaluate the amount of Aβ between timepoints, on day 8, all culture media were replaced with fresh medium. On days 10, 12 and 14, we recovered old media as samples for the ELISA analysis. To check the effect of an oxidant (H_2_O_2_) or anti-oxidant (NAC), all culture media were replaced with fresh medium containing H_2_O_2_ or NAC on day 8. The culture media were subjected to analysis on day 10.

### Enzyme-linked immunosorbent assay (ELISA)

Aβ40 and Aβ42 peptides were quantified using human Aβ40 and Aβ42 commercially available ELISA kits from Immuno-Biological Laboratories (Gumma, Japan). ELISA measurements were performed according to the manufacturer's instructions. Biological triplicates were obtained from supernatants derived from separately differentiated neurons. Technical duplicates were obtained by separating supernatant samples into two wells of primary antibody-conjugated plates.

### Immunofluorescent staining

Cells were washed in D-PBS and then fixed in 4% paraformaldehyde at 4 ºC for 15 min. After washing the cells twice in D-PBS, we incubated the cells for 30 min in 0.025% Triton-10 diluted with blocking reagent (Block Ace, KAC, Kyoto, Japan) at room temperature. Then, primary antibodies were applied overnight at 4 ºC after washing in D-PBS twice. The next day, cells were washed and incubated with secondary antibodies for 1 h at room temperature. Finally, the cells were counterstained with DAPI at room temperature.

The rate of Tbr2-positive cells was calculated using ImageJ software. Using the "analyze particle" function of Image J, we set DAPI positive areas as regions of interest (ROIs). For E-iNs and D-iNs, 69–80 and 69–88 ROIs were counted in each experiment, respectively. The ratio of Tbr2-positive cells to DAPI-positive cells was calculated.

### Quantitative PCR

RNA samples were prepared with the RNeasy Mini Kit (Qiagen, Hilden, Germany) and subjected to reverse transcription with a PrimeScript RT Master Mix (Takara Bio, Shiga, Japan). All procedures were performed following the manufacturer's instructions. Quantitative PCR (qPCR) was performed on the StepOnePlus Real-Time PCR System (Applied Biosystems, Thermo Fisher Scientific, MA, USA). For the detection of transgenes, DNA was subjected to qPCR, and SYBR Premix ExTaqII (Takara Bio, Shiga, Japan) was used for the detection. Data were processed using the ΔΔ cycle threshold method, converted 2^-ΔΔCt^ values, and the relative quantities are shown in the figures. The primer sequences are shown below.

**Table:** 

SOD1 (f)	ACAAAGATGGTGTGGCCGAT
SOD1 (re)	AACGACTTCCAGCGTTTCCT
APP (f)	GACCACTCGACCAGGTTCTG
APP (re)	GCCCACCATGAGTCCAATGA
GAPDH (f)	AATCCCATCACCATCTTCCA
GAPDH (re)	TGGACTCCACGACGTACTCA

### Statistical analysis

GraphPad Prism8 (GraphPad Software, La Jolla, CA, USA) was used for the analysis. All results represent means ± SEM. “n” represents the number of independent cultures. Statistical analysis was performed using Student’s t-test, two-way ANOVA and post-hoc tests. A p value less than 0.05 was considered significant.
